# Editorial: Emerging concepts in *Mycobacterium tuberculosis* pathogenesis: Host-pathogen interaction and stress adaption mechanisms

**DOI:** 10.3389/fcimb.2023.1148756

**Published:** 2023-04-04

**Authors:** Saurabh Pandey, Nasreen Zafar Ehtesham, Seyed Ehtesham Hasnain

**Affiliations:** ^1^ Department of Biochemistry, Jamia Hamdard, New Delhi, India; ^2^ Department of Life Science, School of Basic Sciences and Research, Sharda University, Greater Noida, India; ^3^ Department of Biochemical Engineering and Biotechnology, Indian Institute of Technology-Delhi, New Delhi, India

**Keywords:** host-pathogen interaction, stress adaption, tuberculosis, TB, *Mycobacterium*

The study of host-pathogen interactions is a dynamic and constantly evolving field. Such studies are vital for understanding infectious agent behavior under normal conditions and exposed to host and environmental stresses. The pathogen *Mycobacterium tuberculosis* (*M. tb*) has caused numerous human deaths since prehistoric times. Its stress adaptation mechanisms enable it to subvert host immune challenges. Many *M. tb* encoded effectors exploit host pathophysiology for their benefit. A mechanistic understanding of these effectors will facilitate the design of novel intervention strategies.

In the past decade, next-generation metagenomic sequencing (mNGS) has emerged as a tool capable of identifying rare and difficult-to-diagnose infections, including infections of the central nervous system, blood, and extrapulmonary tuberculosis. The mNGS method offers advantages of short detection times, better sensitivity, and improved specificity over conventional sputum culture techniques. Kong et al. present a case study in which they have documented a skin/cutaneous tuberculosis infection detected by mNGS in wrist wound secretions from a 15-year-old-boy living in Shandong province, China. These authors report that a cure was achieved by anti-TB treatment.

Interferon-induced proteins with tetracopeptides (IFITs), viz. IFIT1, IFIT2, and IFIT3, participate in antiviral immunity by restricting replication. IFIT1 also modulates the Sin3A-HDAC2 transcriptional regulatory complex, which negatively regulates the inflammatory transcription program in LPS-induced macrophages (FEF:10.1016/j.celrep.2018.09.002). Higher levels of IFIT expression reduce *in vitro* survival of distinct drug-sensitive and drug-resistant mycobacteria and correlate with achieving latency in TB infected patients, implying that IFITs are a potential immunotherapeutic route for targeting *M. tb* (Madhvi et al., 2022).

Tumor necrosis factor-alpha (TNF-α) plays an important role in preventing and treating *M. tb* infection and pathogenesis. Pathogenic mycobacteria can regulate host cell TNF-α production, aiding their evasion of anti-TB immune stress. *M. tb*-infected macrophages displayed increased expression of the E3 ubiquitin ligase FBXW7. Specific suppression of FBXW7 with siRNA greatly increased TNF-α expression and eventually cleared infection. In turn, FBXW7 overexpression in RAW264.7 macrophages significantly reduced TNF-α production. Finally, FBXW7 has been shown to reduce TNF-α in a K63-linked ubiquitin signalling dependent manner. In summary, this study revealed that FBXW7 plays a key role in granuloma production that is crucial for *M. tb* pathogenesis. It induces macrophage polarization towards the M2 phenotype and controls TNF-α production *via* a ubiquitination cascade (Song et al., 2022).

The *M. tb* genome encodes the necessary proteins to assemble the Fimbrial low-molecular-weight protein (Flp) type IV pilus. This study investigated the expression of Flp pili-assembly genes (tadZ, tadA, tadB, tadC, flp, tadE, and tadF) in *M. tb* under different growth conditions (exponential, stationary, and hypoxia-induced dormancy), during biofilm formation, and in contact with macrophages. This study revealed that tad/flp gene expression was considerably greater in the stationary phase than that in the other phases. When mycobacteria were propagated in contact with macrophages or epithelial cells for 4 h, gene expression levels were higher than when they were propagated alone in culture media. Overall, these findings indicate that Flp pili genes are expressed during *M. tb* interactions with host cells, indicating a function for Flp pili in host invasion and colonization, boosting bacterial survival during dormancy (Alteri et al., 2022).

Although only a few examples are discussed here, the panoply of host-pathogen interaction and stress adaptation mechanisms of *M. tb* is quite vast. Numerous mechanisms are targeted by mycobacteria-encoded effectors, such as DNA repair, membrane repair, apoptosis, autophagy, innate signalling, and cytokine responses. Moreover, the appearance of highly tolerant persisters in different metabolic states and their role in mycobacterial pathology have yet to be fully elucidated. Another challenging area is the evolution of drug-resistant strains, which may affect TB management regimens, particularly in resource-poor settings. Future research in the field should also take into account the community behavior of the microbial milieu present in immunocompromised individuals, co-infection scenarios, biofilms, quorum sensing programs, and related coordinated responses of the communities or microbial populations coexisting with *M. tb*.

## Author contributions

All authors listed have made a substantial, direct, and intellectual contribution to the work and approved it for publication.

